# The Role of miRNAs as Therapeutic Tools in Sickle Cell Disease

**DOI:** 10.3390/medicina57101106

**Published:** 2021-10-14

**Authors:** Cyril Cyrus

**Affiliations:** Department of Biochemistry, College of Medicine, Imam Abdulrahman Bin Faisal University, Dammam 31141, Saudi Arabia; ccyrus@iau.edu.sa; Tel.: +966-553-241-441

**Keywords:** hemoglobinopathies, miRNA, SCD, HbF, hydroxyurea, globin expression

## Abstract

*Background and Objectives:* Sickle cell disorder (SCD) is a paradigmatic example of a complex monogenic disorder. SCD is characterized by the production of abnormal hemoglobin, primarily in the deoxygenated state, which makes erythrocytes susceptible to intracellular hemoglobin polymerization. Functional studies have affirmed that the dysregulation of miRNAs enhances clinical severity or has an ameliorating effect in SCD. miRNAs can be effectively regulated to reduce the pace of cell cycle progression, to reduce iron levels, to influence hemolysis and oxidative stress, and most importantly, to increase γ-globin gene expression and enhance the effectiveness of hydroxyurea. *Results:* This review highlights the roles played by some key miRNAs in hemoglobinopathies, especially in hematopoiesis, erythroid differentiation, and severity of anemia, which make miRNAs attractive molecular tools for innovative therapeutic approaches. *Conclusions:* In this era of targeted medicine, miRNAs mimics and antagomirs may be promising inducers of HbF synthesis which could ameliorate the clinical severity of SCD.

## 1. Introduction

Sickle cell disorder (SCD), characterized by the production of abnormal hemoglobin, is a paradigmatic example of a monogenic disorder. This hemolytic anemia is a complex disease resulting from a single genetic A/T mutation on codon 6 of the β-globin gene (HBB), which in the deoxygenated state makes erythrocytes vulnerable to intracellular hemoglobin polymerization [1]. Globally, SCD is the most common hemoglobin disorder, with about 7% of the world population being carriers of hemoglobinopathies [2]. SCD (HbSS) individuals display extremely variable clinical severity, ranging from severe anemia, endothelial activation, hemolysis, vascular inflammation, poor development due to repeated vascular occlusion, incidences of acute and chronic sequelae from stroke to multiple end-organ damage [3,4] and failure to thrive. Patients are frequently hospitalized for delayed growth, hand–foot syndrome, fatigue, episodes of chronic pain, frequent infections, and vision problems [5,6].

The primary therapeutic alternatives in SCD consist of transfusion, hydroxyurea and pain management. Hydroxyurea has certain safety concerns, such as myelosuppression and teratogenicity, and is found to be effective only in a few patients. The other recently approved drugs, Crizanlizumab and L-Glutamine, have a modest effect in reducing the incidence of vaso-occlusive crisis episodes among SCD, without improving the hematological parameters [7]. Thus, these drugs neither target the underlying disease pathology nor fully ameliorate disease manifestations [8]. The other alternative is allogeneic bone marrow transplantation. However, less than 20% of SCD patients have related human leukocyte antigen–matched donors. Recently, Frangoul et al., [9] recapitulated the hereditary persistence of fetal hemoglobin phenotype by suppressing BCL11A expression using CRISPR-Cas9 gene-editing techniques in CD34+ hematopoietic stem and progenitor cells at the erythroid-specific enhancer region. The SCD patient receiving autologous CD34+ cells had high levels of allelic editing in blood and bone marrow, elimination of vaso-occlusive episodes and elevated levels of fetal hemoglobin [9]. Even though this approach seems promising, adverse events were reported in the patient, which may need to be considered. The discovery of microRNAs (miRNA) in the mid-90s has changed the dogma of gene expression regulation [10]. miRNAs, an endogenously initiated non-coding class of short RNAs (~22-nt long), are transcribed as precursor molecules that are eventually cleaved by the Dicer and Drosha endoribonucleases [11]. miRNAs post-transcriptionally form a miRNA-mediated silencing complex (miRISC) to inhibit target gene expression, via either translational repression by directly binding to mRNAs, or degradation of target mRNAs by cleavage [11]. miRNAs have been found to play a quintessential role in the regulation of biological processes categorized under epigenetic regulatory mechanisms, such as chromatin-remodeling, proliferation, differentiation, development, homeostasis, and apoptosis [12,13]. Studies in humans have suggested that miRNAs regulate approximately 1/3 of the genome and plausibly >60% of the protein-coding genes [14].

In humans, mature erythrocytes are believed to lack expression of most RNAs [15]. However, these cells still possess abundant and diverse miRNAs. Earlier studies are indicative of a remarkable difference in the miRNA pool between normal and SCD erythrocytes [15,16,17]. This varied miRNA pool in SCD erythrocytes alters the miRNA expression patterns, which may in turn contribute to a reduction in the severity of clinical manifestation in SCD patients. From the initiation of hematopoietic stem cell differentiation to the formation of mature erythrocytes, miRNAs are expressed differentially at various stages of differentiation, and exert effects on the significant transcription factors contributing mainly to fetal hemoglobin (HbF) production. miRNAs also play an essential regulatory role in hematopoietic differentiation, namely erythropoiesis, [18,19] megakaryocytopoiesis, [20] and initiating hematological malignancies [21,22]. Dysregulation of miRNA function is linked to an upsurge in the number of human diseases, predominantly infectious diseases, cancer, and endometrial and hematologic disorders [23]. The function of miRNAs in gene regulation is now well established, and new miRNAs are still being discovered [24].

In blood, miRNAs have been explored in platelets, lymphocytes, monocytes, granulocytes, [25,26] red blood cells (RBCs), [15] and during stages of erythropoiesis [27]. miRNAs have been known to regulate cell-type specific proteins in enucleated cells, such as RBCs and platelets, [15,26] with >200 miRNAs identified in RBCs [15,28]. Significant differences in miRNA expression in mature HbSS (miRNA-299, miRNA-144, miRNA-140, miRNA-451) and HbAA (miRNA-320, let-7s, miRNA-181, miRNA-141) erythrocytes have been noted. The differential expression of erythrocyte miRNA has led to new insights into erythrocyte diseases [15].

**Figure 1:**
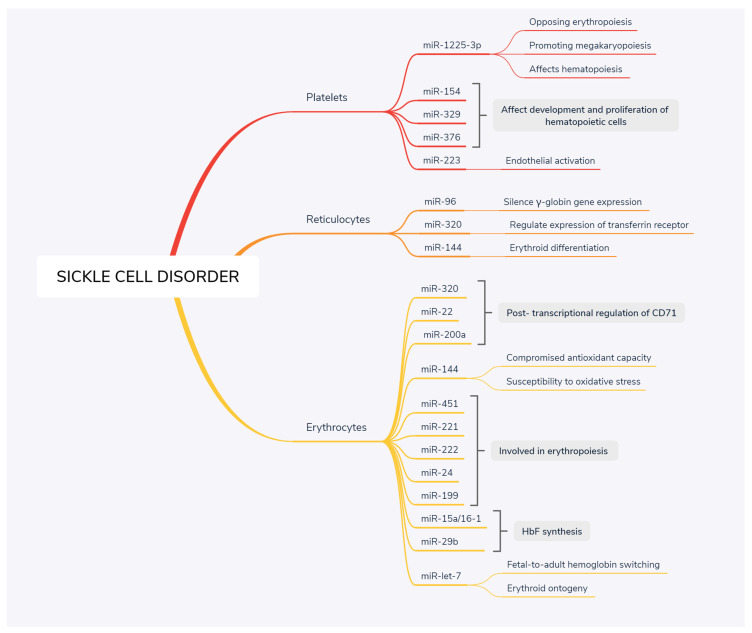
Several key miRNAs associated with sickle cell disease. miRNAs that are specific to erythrocytes, reticulocytes and platelets with dysregulated expression associated with disease manifestation are depicted.

miRNA dysregulation either enhances clinical severity or has an ameliorating effect in SCD. miRNAs can be effectively regulated to reduce the pace of cell cycle progression, to reduce iron levels, to influence hemolysis and oxidative stress, and most importantly, to increase γ-globin gene expression and enhance the effectiveness of hydroxyurea. Hence, the key miRNAs associated (Figure 1) with regulating HbF synthesis, hemolysis-induced irregularity in erythropoiesis, and other ameliorating effects in SCD that may be promising therapeutic targets, are discussed herein.

### 1.1. miRNA-144

The reticulocyte miRNA expression profiling performed in SCD patients with high and low HbF levels identified miRNA-144 to be highly differentially expressed in HbSS cells [29]. Li et al. [29] observed an eightfold up-regulation of miRNA-144–3p and miRNA-144–5p in the low-HbF group compared with the group with elevated HbF levels. The considerably elevated expression of miRNA-144 for the duration of in vitro differentiation of erythroid progenitor cells (HbSS) indicates the possibility of a cell-autonomous phenomenon. The average younger age (earlier developmental stage) of RBCs among SCD patients could also contribute to the dysregulation of miRNA-144 expression. Additionally, the equilibrium of transcription, processing, and decay determines the steady level of miRNA-144 [16,30].

Hemolysis and anemia in SCD can initiate an erythropoietin response and stress erythropoiesis [31], which triggers the expression of the erythropoietin-dependent GATA-1 gene, and in sickle red cell progenitors, an increased generation of miRNA-144 [32]. Sangokoya et al. [16] analyzed erythrocyte miRNAs and identified high erythrocytic miRNA-144 expression to be associated with a severe anemic phenotype. Conversely, NRF2 performs a pivotal role in the cellular antioxidant defense system and oxidative stress response [33]. Increased miRNA-144 expression paves the way for reduced expression of NRF2, thereby leading to a decrease in the cellular levels of glutathione, attenuated antioxidant capacity, and diminished oxidative stress tolerance in HbSS cells. Thereby, the dysregulated regulatory axis of miRNA-144-NRF2 in HbSS cells explains the hemolysis, susceptibility to oxidative stress and compromised antioxidant capacity, decreased hematocrit cell count, and severity of anemia. Additionally, overexpression of miRNA-144 results in a lack of antioxidant proteins, mainly superoxide dismutase 1 (SOD1), catalytic/modifier subunit (GCLC/M), and glutamate–cysteine ligase [16].

Consequent functional analysis of sickle and normal erythroid progenitors has established the ability of miRNA-144 antagomir to facilitate HbF production while increasing NRF2 expression [29]. Hence, manipulation of miRNA-144 expression offers a novel method to reduce the pathological and clinical manifestations of SCD [34].

### 1.2. miRNA-451

miRNA-451 is highly conserved and unique as it does not depend on Dicer, an enzyme required for miRNA maturation. miRNA-451 is exclusively and abundantly present in RBCs [35]. miRNA-451 functions in a cluster with miRNA-144, with this locus regulating the expression of many genes whose products are required for erythropoiesis [32]. During the differentiation of erythroid cells, expression of miRNA-451 becomes elevated [36]. miRNA-451 also contributes to resistance against plasmodium falciparum [37,38] in HbSS erythrocytes. The miRNA-l44/-451 locus, which is critical for erythroid homeostasis under stress conditions, is a key downstream effector of *GATA-1*, and the dysregulation of the miRNA-144/-451 complex triggers a mild form of anemia [13,30,32] Severity of anemia in SCD is associated only with miRNA-144, even though it is part of a polycistronic precursor with miRNA-451. miRNA-451 is more effective in modulating levels of α and β globin, and exerts a less significant effect on γ globin [32,39].

### 1.3. miRNA-29b

In primary erythroid progenitors, the DNA methyltransferase (DNMT) inhibitor miRNA-29b reactivates HBG gene transcription and HbF synthesis. Experimental studies have revealed that miRNA-29b targets one of the chief transcription factors, myeloblastosis oncogene (MYB), that plays a pivotal role in the regulation of γ gene silencing, and is associated with ~40% of inherited HbF variance [40,41]. MYB plays a vital role as an HBG repressor protein, and the overexpression of miRNA-29b to silence MYB could mediate HbF induction. Starlard-Davenport et al. [42] conducted an in vitro study and confirmed the possibility of down-regulation of MYB by miRNA-29b, thereby inducing HbF in KU812 cells. Additive HbF induction was not observed with combined miRNA-29b and hydroxycarbamide treatment in erythroid progenitors generated from adult CD34+ stem cells. 

Furthermore, increased expression of miRNA-29b in reticulocytes isolated from individuals with SCD and elevated HbF levels provides additional evidence for the clinical relevance of this miRNA. For the development of a desirable treatment for SCD, the ability of miRNA-29b to activate HBG transcription and HbF expression in SCD progenitors needs to be evaluated in future studies [42].

### 1.4. miRNA-320

In reticulocytes, miRNA-320 was found to regulate expression of transferrin receptor CD71 [15]. During erythrocyte terminal differentiation, erythrocytic miRNAs interfere directly in the degradation and de-adenylation of mRNA [43,44,45], and the mRNA-targeted blockage of miRNA-320 induces decreased erythrocyte survival.

During reticulocyte terminal differentiation, miRNA-320 plays an important role in down-regulating its target gene, CD71, and persistently high CD71 levels in HbSS cells are associated with modest expression of miRNA-320 [15]. Overexpression of CD71 on the surface of sickle reticulocytes is indicative of a hemolysis-induced irregularity in erythropoiesis [34]. CD71 plays a pivotal role as a transferrin receptor in the terminal differentiation of erythroid cells and in the process of iron absorption. Post-transcriptional regulation of CD71 is regulated by miRNA-22, miRNA-200a, and miRNA-320 [46] To repress CD71 translation, miRNA-320 hybridizes to the 3′UTR of its transcript. The significance of miRNA-320 is made evident by dysregulated maturation and reduced cell survival of sickle cells. The overexpression of the erythropoiesis marker CD71 is reported in many malignancies, and miRNA-320-mediated inhibition of CD71 expression presents a novel treatment strategy to reduce the pace of cell cycle progression and maintain iron levels [15].

### 1.5. Let-7 Family

In humans, the let-7 family of miRNAs mainly targets the heterochronic cascade, which includes the RNA-binding protein LIN28 and is highly regulated during human erythroid ontogeny [47,48]. Lee et al. examined the ability of LIN28B to repress *let-7* miRNA expression, and demonstrated that overexpression of LIN28 or down-regulation of *let-7* miRNAs causes a 19–40% increase in HbF levels in HbSS patients [47].

Noh et al. [28] reported the differential expression of miRNA species among fetal and adult erythroid cells, with a 4.3-to-5.1-fold increase in *let-7* miRNAs. The pattern of up-regulation as compared with down-regulation was contrary to the previously reported [49] pattern of mRNA among the erythroid population in adults, with the pattern being consistent with the general role of miRNA for mRNA degradation. Noh et al. highlighted that the human fetal-to-adult hemoglobin switching phenomenon is associated with up-regulation of the let-7 miRNA group [28]. Azzouzi et al. [50] established that miRNA from the umbilical cord and peripheral blood reticulocytes presented numerous variations in the expression patterns of let-7a.

De Vasconcellos et al. [48] showed that reduction of the let-7 family of miRNAs is sufficient to inhibit B-cell lymphoma 11A (BCL11A) and to enhance HMGA2 for elevated γ globin gene expression [48,51]. As this well-conserved let-7 family has a more generic role, future studies need to provide better insight into the mechanism of the let-7 family in the regulation of erythroid gene activity associated with the fetal-to-adult transition in humans [48].

### 1.6. miRNA-1225-3p

miRNA-1225-3p has a vital role in platelet biology and SCD. Dysregulation of miRNA-1225-3p, which has a significant number of anticipated gene targets, plays a potential biological role in platelet activation. Up-regulation of miRNA-1225-3p lowers PBX homeobox interacting protein 1 (PBXIPI) gene expression. PBXIPI expression plays a vital part in the maintenance of hematopoiesis, and could also affect lineage commitment by opposing erythropoiesis and promoting megakaryopoiesis [52]. Activated platelets contribute to the development of vaso-occlusive disease and intimal damage due to an increase in the adhesion of HbSS RBCs to the endothelium [53] through secretion of fibrinogen, von Willebrand factor [54], TSP-1 [55], transforming growth factor-beta (TGF-β), and platelet-derived growth factor [56], which all promote intimal hyperplasia.

### 1.7. miRNA-221/-222 Cluster

The functional importance of several erythrocyte miRNAs, such as miRNA-221 and miRNA-222, has been emphasized in several studies examining different erythroid differentiation stages [27,57,58]. During the differentiation of erythroid cells, expression of miRNA-221/-222 is reduced [36]. miRNA-221/-222 decreases HbF production by targeting kit receptor ligand (KL) during erythropoiesis [57,59]. miRNA-221/-222 attaches to the 3′UTR to suppress KL, which is capable of reactivating HbF synthesis in normal erythropoiesis and SCD, and reduces proliferation and differentiation of erythropoietic cells [59,60]. Use of antagomiR-221/-222 or exogenous KL therapy helps to increase γ chain production and thereby elevate HbF production for the treatment of patients dependent on HbF reactivation [59].

### 1.8. miRNA-15a/16-1 Cluster

The suppressive effects of miRNA-15a/miRNA-16-1 on chief transcription factors BCL11A, KLF1, and MYB during β globin gene expression can elevate HbF production. Elevated levels of miRNA-15a/16-1 result in down-regulation of an effective negative regulator of HbF expression, MYB factor, thereby inducing delays in hemoglobin switching from fetal to adult and promoting persistent expression of HbF in early erythroid progenitors [40]. The overexpression of miRNA-15a/miRNA-16-1 in Patau syndrome patients was found to down-regulate MYB protein as a mechanism of persistent HbF expression [40]. These studies support the directly targeted physiological role of miRNA-15a/16-1 in the in vivo influence of erythroid progenitors, which suppress MYB, a negative regulator of HBG gene transcription. Therefore, the strategy of inducing changes in the expression of γ globin repressors during erythropoiesis can be effective as a novel therapy for the indirect synthesis of HbF [49,61,62] to improve clinical complications among patients with SCD [63].

### 1.9. miRNA-96

Azzouzi et al. [50] established that the miRNA from the umbilical cord and from peripheral blood reticulocytes have exhibited numerous variations in the expression patterns of miRNA-96. miRNA-96 binds to the ORF of γ-globin mRNA to suppress γ-globin gene expression. During erythropoiesis, elevated expression of miRNA-96 in adult peripheral blood reticulocytes prevents γ-globin mRNA from binding with AGO2 in the miRISC complex, thereby performing an essential role in the regulation of HbF expression [50,63].

### 1.10. miRNA-199

Mnika et al. [33] identified 13 miRNAs that were under-expressed upon exposure to a maximum tolerated dose of hydroxyurea that primarily targeted four genes, namely, Krüppel-like factor 3 (KLF3), Specificity Protein 1 (SP1 Transcription Factor), BCL11A, and MYB, which belong to a common network and were predicted to influence HbF expression and erythropoiesis. The study established that miRNA-199a influenced KLF3 expression [33]. Additionally, key erythroid transcription factors GATA-1 and NF-E2 were regulated by miRNA-199b-5p, a positive erythroid regulator [64].

### 1.11. Other miRNAs

During erythroid maturation, miRNA-26b demonstrates an elevated expression. In patients with hemoglobinopathies, hydroxyurea, which causes HbF augmentation, also exerts an effect on the expression of miRNA-26b [65]. During the initiation of hypoxia-inducible factors under hypoxic conditions, miRNA-210 is overexpressed, which activates the transcription factors in maturing erythroid progenitor cells, thus accelerating the switch to the γ globin gene and thereby establishing the link between erythropoiesis and hypoxia [66,67]. Therefore, miRNA-210 may be an appropriate target for the production of HbF [68,69].

miRNA-486-3p directly inhibits the post-transcriptional regulation of BCL11A expression by binding to its 3′UTR and preventing it from γ gene silencing. Thus, in adult erythropoiesis, miRNA-486-3p contributes to the synthesis of HbF [41,63,70]. miRNA-23a and miRNA-7a are potential inhibitors of both these negative regulators of the globin gene cluster. SP1 inhibition and repression by miRNA-23a increases γ and ε globin expression. KLF3 factor is specifically inhibited by miRNA-27a [71]. Ward et al. have published data to support the role of miRNA-34a in *γ*-globin activation through STAT3 gene silencing [72]. The miRNA expression and its targeted gene associations with different biological effects are summarized in Table 1.

Thus, miRNA mimics and antagomirs of these key miRNAs are promising molecules for therapeutic intervention in hemoglobinopathies [23,42,73]. The identification of the roles played by these miRNAs, mainly in hemoglobin switching and HbF production, has helped in targeting pharmacological miRNA to stimulate HbF production as a novel therapeutic strategy [63]. Hence, miRNAs can replace the use of drugs to improve clinical manifestations and avoid side effects among SCD patients. Due to their capacity to regulate the expression of target genes that cause a wide array of human diseases, the development of miRNA therapeutics is a high priority [42].

## 2. Conclusions

A comprehensive analysis of erythrocytic miRNA expression, along with the approaches available to increase the production of HbF expression, is the primary aim of this review. miRNAs are promising prognostic biomarkers to determine an effective therapeutic regime based on their regulation patterns. As some specific miRNAs are promising inducers of HbF synthesis, which ameliorates the clinical severity of SCD, significant research advancement and commercialization of these miRNAs as therapeutic tools for specific and personalized treatment are in progress. In this present era of targeted medicine, these miRNA-based diagnostic markers and therapeutic tools will move from the laboratory bench to the bedside in the near future.

## Figures and Tables

**Table 1:** miRNA expression and its targeted gene associations with different biological effect in SCD.

microRNA	Target (mRNA or Protein)	Biological Effect	Cell Type	Reference
**Up-Regulated**				
Let-7	*BCL11A*	γ globin gene switching	Erythrocyte	[47]
miRNA-486-3p	*BCL11A*	Increasing expression of γ globin gene	Erythrocyte	[41,63,70]
miRNA-96	γ globin (CDS region)	Suppressing γ globin gene expression	Reticulocyte	[50]
miRNA-29b	*DNMT, MYB*	Increasing expression of γ globin gene	Erythroid progenitor, Reticulocyte	[42]
miRNA-144	*NRF2*	Interference with antioxidant capacity; susceptibility to oxidative stress, hemolysis and severe anemia anemic phenotype	Erythrocyte, Reticulocyte	[16,29]
miRNA-221/-222	*KLFD*	Decreasing of erythroblast proliferation and suppress HbF production	Erythrocyte	[57,59]
miRNA-199a	*KLF3, GATA-1*	Regulating of human erythropoiesis and decrease HbF levels	Erythrocyte	[33,64]
miRNA-451	*GATA-1*	Inducing γ globin gene transcription and suppress α globin, Glycophorin-A	Erythrocyte	[13,30,32]
miRNA-320	CD71	Hemolysis-induced irregularity in erythropoiesis	Reticulocyte	[15,34]
miRNA-1225-3p	*PBXIPI*	Maintenance of hematopoiesis and lineage commitment by opposing erythropoiesis and promoting megakaryopoiesis	Platelet	[52]
miRNA-26b	*GATA1*	Increasing γ globin gene expression	Erythrocyte	[65]
miRNA-210	γ globin	γ globin gene switching	Reticulocyte, Erythrocyte	[66,67]
**Down-Regulated**				
miRNA-23a	*SP1*	Increases γ and ε globin expression	Erythrocyte	[71]
miRNA-27a	*KLF3*	Regulating HbF expression and erythropoiesis	Erythrocyte	[71]
miRNA-34a	*STAT3*	γ-globin activation	Erythrocyte	[72]
miRNA-15a/-16-1	*MYB*	Increasing γ globin gene expression	Erythroid progenitor	[49,61,62]

BCL11A: B-cell lymphoma/leukemia 11A protein, CD71: Transferrin receptor, CDS region; Coding DNA Sequence region mRNA, DNMT: DNA methyltransferase 1, GATA1: GATA-binding factor 1 mRNA, HbF: Fetal hemoglobin, KLF3: Krüppel-like factor 3 mRNA, KLFD: Krüppel-like factor d mRNA, MYB: Proto-oncogene mRNA, NRF2; Nuclear factor (erythroid-derived 2)-like 2 mRNA, PBXIPI: PBX homeobox interacting protein 1, SP1; Specificity protein 1 mRNA, and STAT3: Signal transducer and activator of transcription 3.

## Data Availability

Not Applicable.
